# Supplementary material for: Behavioural individuality determines infection risk in clonal ant colonies
Source: Nat Commun. 2023 Aug 26;14:5233. doi: 10.1038/s41467-023-40983-7 (PMC10460416; doi:10.1038/s41467-023-40983-7)
Supplement: Supplementary file 1 — Supplementary Information [file 41467_2023_40983_MOESM1_ESM.pdf]

Behavioural individuality determines infection risk in clonal ant colonies

Supplementary Information

Author list

Zimai Li[1,2], Bhoomika Bhat[1], Erik T. Frank[3], Thalita Oliveira-Honorato[4], Fumika Azuma[5], Valérie Bachmann[2], Darren J. Parker[6], Thomas Schmitt[3], Evan P. Economo[5], Yuko Ulrich[1,2,4]

Affiliations

[1]Max Planck Institute for Chemical Ecology, Jena, Germany

[2]Institute of Integrative Biology, ETH Zürich, Zürich, Switzerland

[3]Department of Animal Ecology and Tropical Biology, Biocentre, University of Würzburg, Würzburg, Germany

[4]Department of Ecology and Evolution, University of Lausanne, Lausanne, Switzerland

[5]Biodiversity and Biocomplexity Unit, Okinawa Institute of Science and Technology Graduate University, Onna, Japan

[6]School of Natural Sciences, Bangor University, Bangor, United Kingdom

Figures

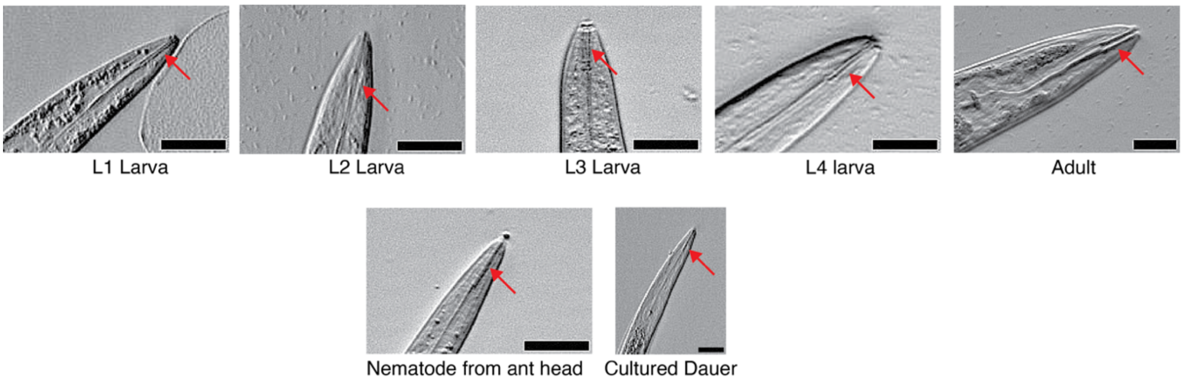

Supplementary Fig. 1 Pharynx morphology of different developmental stages of *Diploscapter* isolated from *O. biroi*. Cultured dauers and nematodes dissected from ant heads have narrower pharynxes than other stages. Red arrows: pharynx. Scale bars: 20 µm.

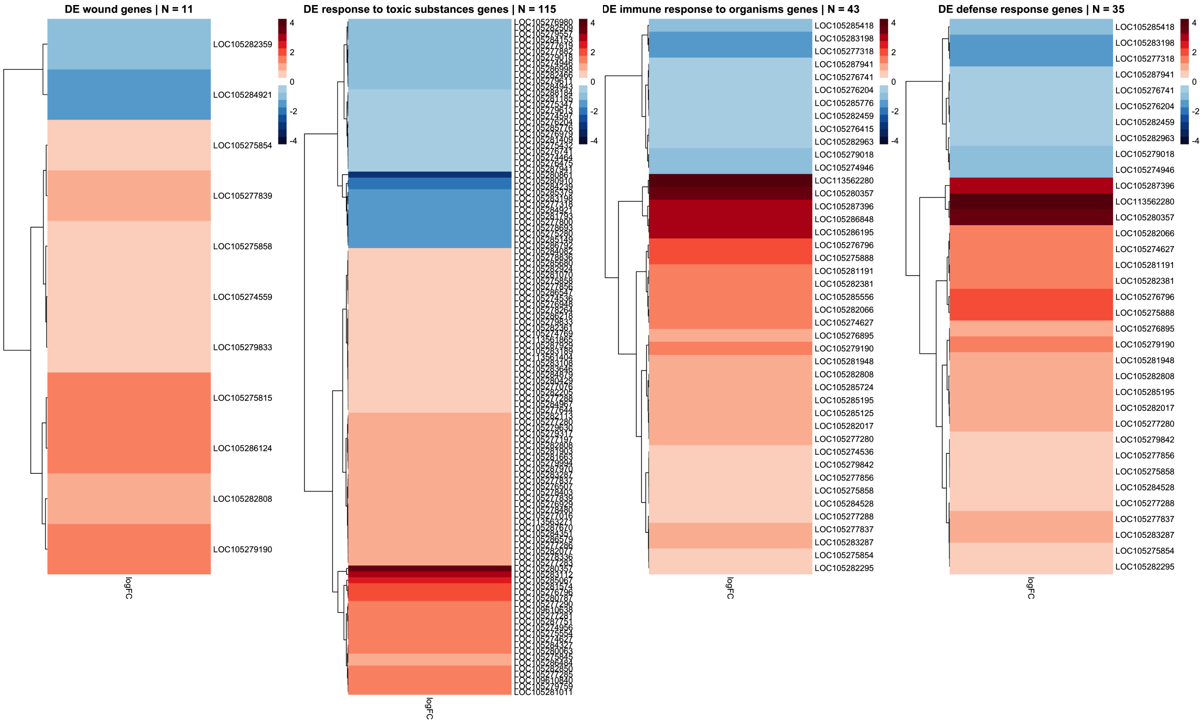

Supplementary Fig. 2 Differential expression of immune gene categories between uninfected and infected PGs. Red: higher expression in infected samples, blue: lower expression.

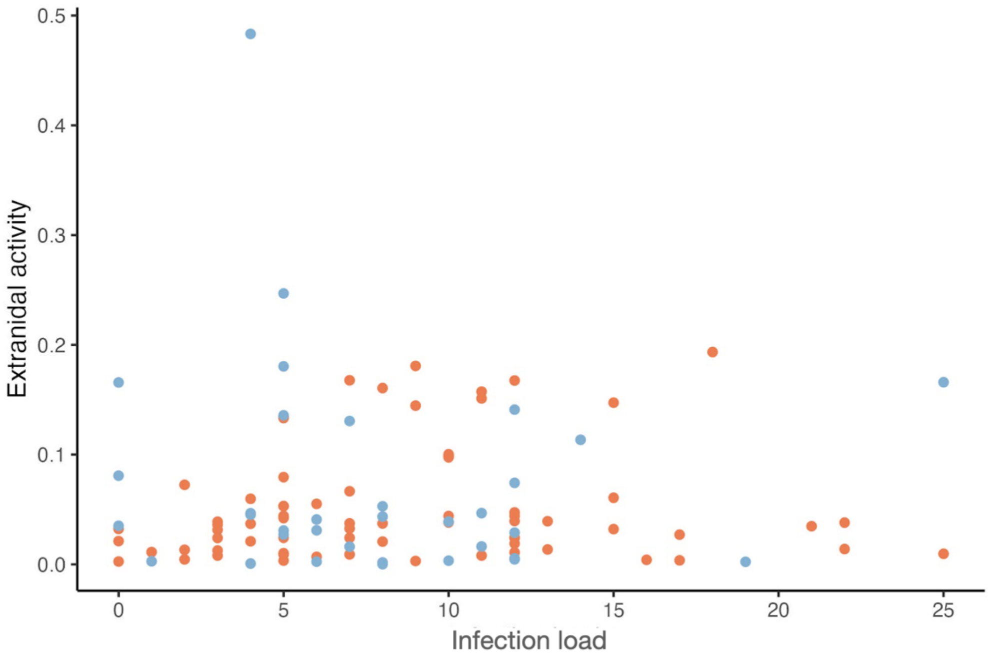

Supplementary Fig. 3 Infection load at the end of the experiment and extranidal activity (proportion of time spent outside the nest) during the first 6 days of the experiment. Dots represent individual infected ants in infected (orange) and mixed (blue) colonies. Source data are provided as a Source Data file.

Supplementary Table 1 Median relative abundance ± SD (%) of all CHCs in ants with different genotypes (B, M) and infection status (uninfected, infected).

Compound	$B_{uninfected}$ (n=10)	$B_{infected}$ (n=10)	$M_{uninfected}$ (n=10)	$M_{infected}$ (n=10)
C25	1.0±0.2	0.4±0.2	1.0±0.2	0.0±0.0
11-MeC25/13-MeC25	3.3±0.4	2.0±0.5	1.6±0.2	2.9±0.8
5-MeC25	1.9±0.5	1.3±0.5	0.6±0.1	1.4±0.6
2-MeC25	7.0±0.4	5.5±0.4	8.4±0.2	4.5±0.5
C26	0.4±0.6	0.3±0.3	0.6±0.1	0.0±0.0
2-MeC26	5.3±0.2	6.5±0.3	3.1±0.2	5.7±0.4
C27	14.2±1.2	9.7±1.1	15.3±1.8	7.5±2.6
13-MeC27	13.1±1.1	12.0±0.2	13.9±0.1	11.5±0.5
11,15-diMeC27	51.8±3.2	59.6±3.2	54.8±1.5	64.0±3.4
3-MeC27	0.0±0.0	0.0±0.0	0.6±0.2	0.7±0.5

Supplementary Table 2 X-ray micro-CT scan parameters.

Species	Specimen identifier	Voxel size	Exposure time (s)	Projections	Voltage (kV)	Power (W)	Source distance (mm)	Detector distance (mm)	Magnification
Lasius niger	CASENT0744001	1.164	22	1601	40	3	-12.504	60.002	4
Ooceraea biroi	CASENT0741215	0.995	13	3201	40	3	-9.516	55.019	4
Paratrechina longicornis	CASENT0744371	0.778	24.5	1601	40	3	-10.043	77.037	4

Supplementary Table 3 Read counts and accession numbers for sequenced samples.

Sample Name	Tissue	Infection status	Raw reads	Trimmed reads	Mapped reads	SRA accession
Obir_BRN_I_rep2	Brain	Infected	20289459	19398729	13354218	SRR17285162
Obir_BRN_I_rep4	Brain	Infected	18353702	17586526	12783548	SRR17285154
Obir_BRN_I_rep6	Brain	Infected	17616117	16902695	12235276	SRR17285148
Obir_BRN_I_rep8	Brain	Infected	24303035	22959320	15826010	SRR17285160
Obir_BRN_NI_rep1	Brain	Uninfected	22676153	21646247	15233311	SRR17285163
Obir_BRN_NI_rep3	Brain	Uninfected	20565461	19756221	14353756	SRR17285155
Obir_BRN_NI_rep5	Brain	Uninfected	24824020	23803884	17407569	SRR17285149
Obir_BRN_NI_rep7	Brain	Uninfected	24411277	23512768	17719564	SRR17285161
Obir_PPG_I_rep2	PG	Infected	18715309	17973940	12478900	SRR17285150
Obir_PPG_I_rep4	PG	Infected	18754300	18090812	12187685	SRR17285153
Obir_PPG_I_rep6	PG	Infected	20491749	19149551	13668256	SRR17285158
Obir_PPG_I_rep8	PG	Infected	18310898	17618160	13177067	SRR17285156
Obir_PPG_NI_rep1	PG	Uninfected	19101715	18383613	14485763	SRR17285151
Obir_PPG_NI_rep3	PG	Uninfected	16605032	15979022	12686796	SRR17285152
Obir_PPG_NI_rep5	PG	Uninfected	17770008	16957202	13142744	SRR17285159
Obir_PPG_NI_rep7	PG	Uninfected	20854374	19986375	15554046	SRR17285157
