# Supplementary material for: Behavioural individuality determines infection risk in clonal ant colonies
Source: Nat Commun. 2023 Aug 26;14:5233. doi: 10.1038/s41467-023-40983-7 (PMC10460416; doi:10.1038/s41467-023-40983-7)
Supplement: Supplementary file 3 — Description of Additional Supplementary Files [file 41467_2023_40983_MOESM3_ESM.pdf]

Description of Additional Supplementary Files

File Name: Supplementary Data 1
Description: Differential expression analysis of genes in the pharyngeal gland

File Name: Supplementary Data 2
Description: Differential expression analysis of genes in the brain

File Name: Supplementary Data 3
Description: Gene Ontology term enrichment analysis of the differentially expressed genes in the pharyngeal gland

File Name: Supplementary Movie 1
Description: Nictating nematode attaches to a clonal raider ant
