# Supplementary material for: Behavioural individuality determines infection risk in clonal ant colonies
Source: Nat Commun. 2023 Aug 26;14:5233. doi: 10.1038/s41467-023-40983-7 (PMC10460416; doi:10.1038/s41467-023-40983-7)
Supplement: Supplementary file 8 — Reporting Summary [file 41467_2023_40983_MOESM8_ESM.pdf]

# nature portfolio

Corresponding author(s): Yuko Ulrich

Last updated by author(s): Aug 9, 2023



# Reporting Summary

Nature Portfolio wishes to improve the reproducibility of the work that we publish. This form provides structure for consistency and transparency in reporting. For further information on Nature Portfolio policies, see our Editorial Policies and the Editorial Policy Checklist.

## Statistics

For all statistical analyses, confirm that the following items are present in the figure legend, table legend, main text, or Methods section.

| n/a | Confirmed | |
|---|---|---|
| ☐ | ☒ | The exact sample size (*n*) for each experimental group/condition, given as a discrete number and unit of measurement |
| ☒ | ☐ | A statement on whether measurements were taken from distinct samples or whether the same sample was measured repeatedly |
| ☐ | ☒ | The statistical test(s) used AND whether they are one- or two-sided<br>*Only common tests should be described solely by name; describe more complex techniques in the Methods section.* |
| ☐ | ☒ | A description of all covariates tested |
| ☐ | ☒ | A description of any assumptions or corrections, such as tests of normality and adjustment for multiple comparisons |
| ☐ | ☒ | A full description of the statistical parameters including central tendency (e.g. means) or other basic estimates (e.g. regression coefficient) AND variation (e.g. standard deviation) or associated estimates of uncertainty (e.g. confidence intervals) |
| ☐ | ☒ | For null hypothesis testing, the test statistic (e.g. *F*, *t*, *r*) with confidence intervals, effect sizes, degrees of freedom and *P* value noted<br>*Give P values as exact values whenever suitable.* |
| ☒ | ☐ | For Bayesian analysis, information on the choice of priors and Markov chain Monte Carlo settings |
| ☐ | ☒ | For hierarchical and complex designs, identification of the appropriate level for tests and full reporting of outcomes |
| ☒ | ☐ | Estimates of effect sizes (e.g. Cohen's *d*, Pearson's *r*), indicating how they were calculated |

*Our web collection on statistics for biologists contains articles on many of the points above.*

## Software and code

Policy information about availability of computer code

| | |
|---|---|
| Data collection | Micro-CT data were collected with software Amira (v2019.2) and VGStudio (v3.4). Chemical (CHC) data was collected using ChemStation (v. F.01.03.2357) was used. Nematode imaging was performed with ZEISS Scout-and-Scan Control System Reconstructor and Zeiss Zen Blue. Custom MATLAB (v.2022a) software was used to acquire videos for behavioural tracking. |
| Data analysis | For phylogenetic analyses, Geneious Prime (v. 2021.0.3), PRANK (v.100802), Gblocks (v. 0.91), and RAxML (v. 8.2.12) were used. For transcriptomic analyses, Trimmomatic (v. 0.36), Kallisto (v. 0.46.0), tximport (v. 1.10.1), and Blast2GO were used. Behavioural tracking analyses were performed using anTraX (v. 1.0.2). Standard statistical tests were performed in R (v4.0.3 & v. 4.1.2). All custom scripts used in this analysis are deposited in the following Edmond repository (https://doi.org/10.17617/3.16NKXM). |

For manuscripts utilizing custom algorithms or software that are central to the research but not yet described in published literature, software must be made available to editors and reviewers. We strongly encourage code deposition in a community repository (e.g. GitHub). See the Nature Portfolio guidelines for submitting code & software for further information.

## Data

Policy information about availability of data

All manuscripts must include a data availability statement. This statement should provide the following information, where applicable:

- Accession codes, unique identifiers, or web links for publicly available datasets
- A description of any restrictions on data availability
- For clinical datasets or third party data, please ensure that the statement adheres to our policy

Behavioural and chemical data generated in this study are available in the following Edmond repository (https://doi.org/10.17617/3.16NKXM). RNA-sequencing data are available at NCBI's sequence read archive under the following BioProject accession: PRJNA791185. RNA-sequencing accession numbers can be found in Supplementary Table 6.

## Research involving human participants, their data, or biological material

Policy information about studies with human participants or human data. See also policy information about sex, gender (identity/presentation), and sexual orientation and race, ethnicity and racism.

| | |
|---|---|
| Reporting on sex and gender | n.a. |
| Reporting on race, ethnicity, or other socially relevant groupings | n.a. |
| Population characteristics | n.a. |
| Recruitment | n.a. |
| Ethics oversight | n.a. |

Note that full information on the approval of the study protocol must also be provided in the manuscript.

# Field-specific reporting

Please select the one below that is the best fit for your research. If you are not sure, read the appropriate sections before making your selection.

☒ Life sciences ☐ Behavioural & social sciences ☐ Ecological, evolutionary & environmental sciences

For a reference copy of the document with all sections, see nature.com/documents/nr-reporting-summary-flat.pdf

# Life sciences study design

All studies must disclose on these points even when the disclosure is negative.

| | |
|---|---|
| Sample size | No statistical tools were used to predetermine sample sizes. Sample sizes (number of ants per colony and number of colonies per treatment) were chosen based on previous work* showing that these sample sizes provided sufficient power to detect behavioural differences when those differences exist.<br>*Ulrich, Y., Saragosti, J., Tokita, C. K., Tarnita, C. E. & Kronauer, D. J. C. Fitness benefits and emergent division of labour at the onset of group living. Nature 560, 635–638 (2018). |
| Data exclusions | One genotype (A) was excluded from the chemical (CHC) analysis because ants in the uninfected colony were found to be contaminated and we could not estimate the effect of infection on CHC profiles in this genotype. |
| Replication | For experiments on the effects of infection on survival, effects of behaviour on infection, and effects of infection of behaviour, experimental replicate colonies were used (and the effect of colony taken into account in statistical analyses). The experiment on the effects of infection on CHC profiles was performed in two independent clonal raider ant genotypes. |
| Randomization | Individual ants were randomly collected from stock colonies and assigned to pooled samples/treatments/experimental colonies at random. The position of colonies in the tracking setup was randomized. |
| Blinding | Investigators did not directly participate in the acquisition of behavioural, sequencing and chemical data because these processes were automated. |

# Reporting for specific materials, systems and methods

We require information from authors about some types of materials, experimental systems and methods used in many studies. Here, indicate whether each material, system or method listed is relevant to your study. If you are not sure if a list item applies to your research, read the appropriate section before selecting a response.

## Materials & experimental systems

| n/a | Involved in the study |
| --- | --- |
| ☒ | ☐ Antibodies |
| ☒ | ☐ Eukaryotic cell lines |
| ☒ | ☐ Palaeontology and archaeology |
| ☐ | ☒ Animals and other organisms |
| ☒ | ☐ Clinical data |
| ☒ | ☐ Dual use research of concern |
| ☒ | ☐ Plants |

## Methods

| n/a | Involved in the study |
| --- | --- |
| ☒ | ☐ ChIP-seq |
| ☒ | ☐ Flow cytometry |
| ☒ | ☐ MRI-based neuroimaging |

# Animals and other research organisms

Policy information about studies involving animals; ARRIVE guidelines recommended for reporting animal research, and Sex and Gender in Research

| | |
| --- | --- |
| Laboratory animals | Ants:<br>- Clonal raider ants, Ooceraea biroi, workers of clonal lines A, B, and M. Age of animals: 5-days old larvae to ca. six months old adults.<br>- Black garden ant, Lasius niger, worker of unknown age and genotype<br>- Longhorn crazy ant, Paratrechnia longicornis, worker of unknown age and genotype<br><br>Nematodes:<br>- Diploscapter sp. isolated from O. biroi<br>- Diploscapter sp. isolated from L. niger<br>- Diploscapter sp. isolated from P. longicornis |
| Wild animals | No wild animals were used in the study |
| Reporting on sex | All ants were female; sex of nematodes is unknown |
| Field-collected samples | No field-collected samples were used in the study |
| Ethics oversight | No ethical guidance or approval was required because this research deals with invertebrates (insects and nematodes) that are not regulated in this respect. |

Note that full information on the approval of the study protocol must also be provided in the manuscript.
